# Special Issue “Advances in Kinanthropometry: Techniques and Applications in Sports and Health”

**DOI:** 10.3390/jfmk11010056

**Published:** 2026-01-29

**Authors:** Stefania Toselli, Natascia Rinaldo, Luciana Zaccagni

**Affiliations:** 1 Department for Life Quality Studies, University of Bologna, 47921 Rimini, Italy; stefania.toselli@unibo.it; 2 Department of Neuroscience and Rehabilitation, Faculty of Medicine, Pharmacy and Prevention, University of Ferrara, 44121 Ferrara, Italy; natascia.rinaldo@unife.it

## 1. Background and Current Challenges in Kinanthropometry

The field of kinanthropometry has developed substantially over recent years, reflecting the growing interest in understanding human structure, function, and performance through methods that capture the multidimensional nature of growth, training, health, and aging [1]. While traditional anthropometric measurements remain essential, they are now supported by technological innovations, computational approaches, and integrative perspectives that align morphology with neuromuscular function, cognition, metabolism, and behavior. This evolution is driven by an increasingly complex scientific landscape in which sport performance, health assessment and monitoring, and biological development can no longer be examined in isolation.

Although significant progress has been made in the study of kinanthropometry, notable gaps remain across multiple areas of the field. Updated population-specific morphological profiling tools are necessary, particularly in sports, in order to address differences in anthropometric characteristics. Accurately evaluating growth and maturation during childhood and adolescence remains challenging, as biological variability increases and the existing prediction tools do not always perform consistently across all populations. In sports science, understanding how structural traits interact with neuromuscular and cognitive variables to influence performance requires the development of more integrative frameworks. In public health, accessible and reliable methods for monitoring body composition and identifying sarcopenia or obesity remain a priority, particularly in low-resource settings. Moreover, additional research is necessary for special populations, including para-athletes, preschool children, and aging individuals, who often remain underrepresented in anthropometric investigations.

This Special Issue, “Advances in Kinanthropometry: Techniques and Applications in Sports and Health”, addresses these challenges and emphasizes the importance of multidisciplinary work. The papers included offer an updated and comprehensive overview of contemporary research, covering methodological innovations, sport-specific profiling, developmental trajectories, health-related analyses, and systematic syntheses of evidence.

## 2. Advances in Methods and Sport-Specific Applications

A major direction emerging from this Special Issue is the incorporation of neuromotor and cognitive indicators into morphological profiling. Carvalho et al. provide an innovative perspective by examining the associations between neurofeedback outcomes and anthropometric, physical, technical, and tactical performance in young women football players. Their approach demonstrates how neural activity and cognitive processing may be combined with structural assessments to obtain a more complete representation of performance determinants. This expanded perspective illustrates the potential for future applications of kinanthropometry in models that incorporate central nervous system functioning alongside somatic traits, especially in team sports where decision-making and perceptual–motor coordination play a crucial role.

The importance of sport-specific morphological profiling is reinforced in several studies. Ojeda-Aravena et al. describe sex-based differences in young basketball players, revealing variations in neuromuscular and structural characteristics that are already substantial by mid-adolescence. These findings are essential for designing tailored training programs that consider maturation timing and sex-specific developmental pathways.

Similarly, De la Rosa et al. provide detailed anthropometric and neuromuscular profiles for male volleyball players, focusing on positional differences that significantly influence performance demands. Their results demonstrate the practical value of integrating anthropometric, baropodometric, and handgrip strength assessments into sport selection and training design.

The longitudinal case study by Carvajal-Veitía et al. documents morphological adaptations in a five-time Olympic Greco-Roman wrestling champion preparing for the Paris 2024 Games. Longitudinal data of this nature are rare in research on elite sports, particularly for athletes with extended careers at the highest competitive level. The study not only illustrates the dynamic nature of elite performance but also highlights the critical role of anthropometric monitoring in weight category sports, where body composition management is a cornerstone of competitive success.

The Special Issue further underscores the relevance of studying body asymmetry in sports characterized by unilateral loading. Herrera-Amante et al. investigate adolescent canoeists and kayakers, demonstrating structural and functional directional biases that reflect sport-specific demands. Their findings support the importance of monitoring asymmetry to prevent injuries and optimize performance, aligning with current trends in injury prevention research.

Large-scale studies remain essential for defining normative values across different populations. The extensive contribution by Martínez-Mireles et al. provides a comprehensive somatotype dataset covering 43 sports and a large national sample of Mexican athletes. This research is fundamental for understanding population-level variations and for contextualizing athlete selection and training design within specific cultural and environmental settings.

## 3. Growth, Development, and Health-Oriented Perspectives

In addition to performance in sports, this Special Issue includes an important series of papers on growth, maturation, and development during childhood and adolescence, periods in which anthropometric variability is particularly pronounced. Gerber and Pienaar show that the Body Mass Index (BMI) is often insufficient for capturing meaningful changes in children’s body composition, reinforcing the need for additional indicators, especially during periods of rapid growth.

Zaccagni et al. study adolescent soccer players and examine how the physical changes induced by training interact with body image perception—an aspect that is too often overlooked but that may influence self-esteem, motivation, adherence to training, and overall well-being.

The methodological study by Cular et al. comparing different maturity prediction methods illustrates the persistence of inconsistencies between models. The increasing adoption of new technologies such as the BAUSport™ SonicBone system requires careful validation, especially when used for talent identification, training prescription, or injury risk estimation in youth athletes.

The Special Issue also includes studies exploring early childhood. Kapo-Gurda et al. reveal posture differences between preschool boys and girls, emphasizing that musculoskeletal development begins early and requires monitoring to prevent future postural deviations. Meanwhile, Guzmán-Muñoz et al. provide insight into the relationship between body composition and proprioception in children, illustrating how excess adiposity may impair sensory–motor functioning and potentially influence motor skill development.

The studies included in this Special Issue additionally address kinanthropometry’s relevance to public health. Campoli et al. examine behavioral and lifestyle profiles across obesity classes in a large Italian clinical cohort, showing a clear heterogeneity in physical activity patterns, dietary preferences, and behavioral traits. These findings support the need for individualized approaches to obesity interventions and contribute to increasing evidence that anthropometric classifications alone cannot adequately describe health risks.

The application of machine learning marks another noteworthy innovation. Forte et al. used metabolic, anthropometric, and physical fitness variables to model sleep quality in older adults. Their findings illustrate how multidimensional models can help identify predictors of health-related outcomes that are influenced by complex physiological interactions.

Likewise, González-Martin et al. propose a machine learning model that accurately predicts low appendicular lean mass, offering a cost-effective approach to sarcopenia screening. This is particularly relevant in settings where advanced imaging tools such as DXA are unavailable.

The inclusion of research on underrepresented populations is another strength of this Special Issue. Becerra-Patiño et al. conducted a systematic review of morphological differences across playing positions in blind five-a-side soccer players, a sport that requires unique perceptual and motor adaptations. This work highlights gaps in parasport research and encourages more inclusive efforts to understand morphological and functional factors in athletes with disabilities.

## 4. Future Directions and Conclusions

Collectively, the papers included in this Special Issue address the main knowledge gaps identified in the field. They provide updated reference data from multiple countries, propose new approaches for linking structure and function, evaluate methodological tools for assessing growth and maturation, include new computational approaches for analyzing body composition and performance predictors, and investigate populations ranging from preschool children to elite adult athletes and older adults. This diversity of research questions and approaches reflects the vitality of kinanthropometry as a contemporary scientific discipline.

There are several significant future directions for the study of kinanthropometry. Longitudinal studies remain essential for understanding how morphological, neuromuscular, and metabolic traits evolve over time, particularly during critical developmental phases and in high-performance contexts. Future research must also prioritize validating and standardizing new technologies, especially portable imaging devices, 3D scanning systems, and automated measurement tools, to ensure that innovations are reliable, replicable, and suitable for use across different populations and settings.

In addition, the integration of anthropometric, neuromuscular, metabolic, psychological, and behavioral data will increasingly support multidimensional models that are able to capture the complexity of human performance and health. Cross-cultural research will remain crucial for developing more representative global reference values and reducing biases in diagnoses and performance assessment. The application of kinanthropometry in public health is also essential, particularly for the development of accessible tools for obesity and sarcopenia screening. Finally, future research should prioritize the inclusion of underrepresented groups—including para-athletes, preschool children, and individuals from low- and middle-income countries—to provide a more comprehensive understanding of human morphology.

In summary, this first volume of the Special Issue “Advances in Kinanthropometry: Techniques and Applications in Sports and Health” offers an extensive and diverse representation of the current and emerging directions of study in the field of kinanthropometry. Through methodological innovations, sport profiling, developmental analyses, and health-oriented research, the contributors to the Special Issue help advance kinanthropometry as a fundamental tool in both applied and theoretical contexts. The findings presented here will help to stimulate further interdisciplinary collaborations and will contribute to the growing recognition of kinanthropometry as an essential discipline for understanding human development, performance, and health.
